# Improvement of Intramuscular Fat in *longissimus* Muscle of Finishing Thai Crossbred Black Pigs by Perilla Cake Supplementation in a Low-Lysine Diet

**DOI:** 10.3390/foods11070907

**Published:** 2022-03-22

**Authors:** Korawan Sringarm, Niraporn Chaiwang, Watcharapong Wattanakul, Prapas Mahinchai, Apinya Satsook, Rakkiat Norkeaw, Mintra Seel-audom, Tossapol Moonmanee, Supamit Mekchay, Sarana Rose Sommano, Warintorn Ruksiriwanich, Pornchai Rachtanapun, Kittisak Jantanasakulwong, Chaiwat Arjin

**Affiliations:** 1 Department of Animal and Aquatic Sciences, Faculty of Agriculture, Chiang Mai University, Chiang Mai 50200, Thailand; korawan.s@cmu.ac.th (K.S.); Apinya_sat@cmu.ac.th (A.S.); rakkiat_nor@cmu.ac.th (R.N.); mintra.s@cmu.ac.th (M.S.-a.); tossapol.m@cmu.ac.th (T.M.); supamit.m@cmu.ac.th (S.M.); 2 Cluster of Research and Development of Pharmaceutical and Natural Products Innovation for Human or Animal, Chiang Mai University, Chiang Mai 50200, Thailand; sarana.s@cmu.ac.th (S.R.S.); warintorn.ruksiri@cmu.ac.th (W.R.); 3 Cluster of Agro Bio-Circular-Green Industry (Agro BCG), Chiang Mai University, Chiang Mai 50200, Thailand; pornchai.r@cmu.ac.th (P.R.); kittisak.jan@cmu.ac.th (K.J.); 4 Department of Agricultural Technology and Development, Faculty of Agricultural Technology, Chiang Mai Rajabhat University, Chiang Mai 50300, Thailand; niraporn_cha@g.cmru.ac.th (N.C.); w.wattanakul@rocketmail.com (W.W.); 5 Chiang Mai Livestock Research and Breeding Center, Department of Livestock Development, Chiang Mai 50120, Thailand; prapasdld@hotmail.com; 6 Department of Plant and Soil Sciences, Faculty of Agriculture, Chiang Mai University, Chiang Mai 50200, Thailand; 7 Department of Pharmaceutical Sciences, Faculty of Pharmacy, Chiang Mai University, Chiang Mai 50200, Thailand; 8 School of Agro-Industry, Faculty of Agro-Industry, Chiang Mai University, Chiang Mai 50100, Thailand

**Keywords:** perilla cake, finishing pig, Thai crossbred pig, meat quality, low-lysine diet, intramuscular fat, fatty acid profile

## Abstract

This study was conducted to find out the effects of perilla cake (PC) supplementation in a low-lysine diet on Thai crossbred finishing pigs’ productivity, carcass and meat quality, and fatty acid composition. For six weeks, a total of 21 barrows of finishing pigs were fed with three dietary treatments (T1: basal diet, T2: 2.5 percent PC supplementation in a low-lysine diet, and T3: 4.5 percent PC supplementation in a low-lysine diet). The results show that the intramuscular fat and marbling score was significantly increased by T2 and T3. On the other hand, it was found that the boiling loss and shear force value were significantly decreased by T2 and T3 (*p* < 0.05). In a low-lysine diet, dietary PC supplementation caused a significant increase in malondialdehyde levels in meat (*p* < 0.05) compared with the basal diet. It was also shown that alpha-linolenic acid level in backfat and the *longissimus thoracis et lumborum* muscle was increased considerably by T2 and T3. Therefore, supplementing PC in a low-lysine diet may be an alternative strategy for improving the meat quality of late-phase pigs.

## 1. Introduction

Pig production is critical to Thailand’s food chain as the primary source of protein. Additionally, pig production has increased dramatically during the last decade [1]. Commercial breeds such as the Large White, Landrace, Duroc, and crosses of these are the main breeds in this production system. Likewise, native pigs or breeds of indigenous and exotic races have been raised, including ‘Rad’, ‘Phuang’, and ‘Kwai’ [2]. These genotypes are important to smallholder farming and highland agriculture in Thailand. However, the limitation of native pig breeds is that they grow slowly and their reproduction rates are lower than those of commercial breeds. On the other hand, they are well adapted to hot and humid climates and to low-quality feed [1,2]. As a result, various studies have been conducted to improve indigenous pigs’ productivity and meat quality through crossbreeding with commercial breeds such as Duroc, Large White, Pietrain, etc. [3,4,5,6] to respond to consumer demand. Furthermore, Glinoubol et al. [5] reported that the Thai native pigs are known for their high intramuscular fat content. Therefore, it is interesting to study the potential of Thai crossbred pigs, especially the indicators of meat quality, such as intramuscular fat.

In recent years, a trend in pig production has been toward higher-quality pork. The intramuscular fat (IMF) content of pork is a critical factor in determining consumer acceptance. The critical elements for producing high-quality pork with a high IMF content are achieved through breeding selection and nutritional management [7]. Numerous studies have been conducted to demonstrate that nutritional control strategies, specifically a reduced lysine level in a low-protein diet, had an effect on the accumulation of IMF in finisher pigs [7,8,9,10,11]. Even so, there was concern that lysine- or protein-deficient diets may have an adverse effect on pig productivity. However, previous studies indicated that a lysine shortage in the diet had no effect on pigs’ productive performance [7,11]. Additionally, the rise in IMF content should be followed by an increase in high-quality fat in pig muscle. The fatty acid composition of the IMF has a significant impact on the quality aspects of pork processing and the nutritional value of pork for customers. This may be related to the inclusion of feedstuffs containing high-quality fatty acids to the pig diet. Functional substances can be added to feed to improve the n-3 fatty acid composition of pork. In a previous work, Okrouhlá et al. [12] reported that pigs fed a diet supplemented with linseed exhibited a significantly increased content of polyunsaturated fatty acids (PUFAs), especially n-3 PUFAs. Tonnac and Mourot [13] also reported that the content of n-3 PUFA content of pigs’ *longissimus* muscle and subcutaneous backfat increased in response to a dietary intake of linseed and microalgae rich in docosahexaenoic acid (DHA).

Perilla (*Perilla frutescens*) is a Lamiaceae/Labiatae family member [14]. It is extensively cultivated in Asian countries including China, Korea, India, and Thailand. Perilla is frequently utilized in cooking as a source of edible oil. Additionally, it has biological activities, for example antiviral, anti-inflammatory, and antioxidant activities. [15,16,17]. Thailand produces approximately 272 tons of perilla seeds per year for perilla oil production, with approximately 60% of the by-products being perilla meal [18,19]. Perilla cake (PC) is a by-product of perilla seeds in the oil refinery industries via screw pressing. [18]. PC is a rich source of PUFA, particularly alpha-linolenic acid (ALA) (55.97%), with a 10.52% ether extract (EE) content [18]. Arjin et al. [20] explained that perilla cake with a high ALA content might have potential as a feed supplement to enhance n-3 PUFA levels in pig tissues. For this reason, PC is interesting to consider as an alternative feedstuff for increasing IMF accumulation and modifying the fatty acid profile in pigs. In several studies, perilla meal was used to improve the fatty acid content in livestock. Hadi and Sudiyano [21] showed that dietary supplementation of with perilla seed meal in ducks improved omega-3 fatty acid levels (0.94%). In addition, dietary supplementation of perilla oil in broilers results in greater levels of ALA, DHA, PUFA, and n-3 fatty acids [22]. Moreover, growing pigs that received perilla cake supplementation in diet significantly increased the ALA content, whereas the n-6/n-3 ratio decreased significantly in the *longissimus* muscle, backfat, and abdominal fat [20]. However, no study has been conducted to determine the efficacy of supplementing PC in finishing pig diets with lysine shortage in order to improve IMF and fatty acid profiles. We postulate that lowering lysine consumption promotes IMF formation in late-stage pig muscle and that supplementing with PC improves the fatty acid composition. Therefore, the purpose of this study is to determine the influence of PC supplementation in low-lysine feed on the productive performance, meat quality, and fatty acid profile of finishing crossbred pigs.

## 2. Materials and Methods

### 2.1. Pigs and Management

A total of 21 barrow four-line Thai crossbred pigs ((Meishan × Duroc) × (Thai native × Pietran)) with an average initial body weight (BW) of 73.9 ± 3.3 kg were randomized into three dietary treatments consisting of seven pigs per treatment. Individual pigs were housed in 2.0 m^2^ areas on concrete flooring within the same housing in the Mae Hia Agricultural Research, Demonstration and Training Center, Chiang Mai University (CMU) for 6 weeks of the trial period. Pigs were acclimatized to feed and housing for 15 days, including deworming and vaccination, before the commencement date of the treatment. All pigs were allowed free access to feed and water.

### 2.2. Diet

Maize, broken rice, rice bran, fish meal, and soybean meal were the main components of the three dietary formulations used in this investigation. The treatments were as follows: basal diet (control/T1), low-lysine level basal diet and supplementation of PC at 2.5% (T2), and low-lysine basal diet and supplementation of PC at 4.5% (T3). According to the National Research Council (NRC) recommendations, the feed was designed to have equal amounts of crude protein, vitamin, mineral, and metabolizable energy to suit the requirements of finishing pigs [23]. The composition, nutritional values, and fatty acid profiles of each diet are shown in Table 1 and Table 2. The lysine and methionine in the diet was measured using Capel-205 capillary electrophoresis (Lumex Instruments, St. Petersburg, Russia), according to Komarova et al. [24]. Throughout the trial period, daily feed intake *ad libitum* and weekly body weight measures were utilized to assess productive performance, which included average daily gain (ADG), average daily feed intake (ADFI), and feed conversion ratio (FCR).

### 2.3. Slaughter Procedure

At the end of the experimental period, all the pigs were fasted for 12 h before slaughter. All experimental procedures were conducted in accordance with the good manufacturing practices of the abattoir Thai Agricultural Standard TAS 9004-2004 [25]. Prior to transfer to the Huay Kaew Slaughterhouse, Chiang Mai, Thailand (a 10 km journey), the pigs were individually weighed at the farm. Electrical stunning and exsanguination were used to euthanize the pigs. The internal organs were collected and emptied. The carcasses were separated, and half carcass was weighed for the hot carcass weight. Then, all carcasses were chilled at 4 °C for 24 h.

### 2.4. Evaluation of Carcass Characteristics and Tissue Sampling

The carcasses were weighed 24 h after chilling at 4 °C to obtain the chilled carcass weight. The carcass dressing percentages were determined by dividing the carcass weight by its fasting weight. The carcass length according to Álvarez-Rodríguez and Teixeira [26], The thickness of the backfat was determined at the 11th rib (along with the skin) by using a vernier caliper on the *longissimus thoracis et lumborum* (LTL) muscle in centimeters. Carcasses were prepared into four lean cuts following Thai and USDA standards [3]: boston, loin, picnic, and ham. The backfat and LTL muscle were harvested from the right side of the carcass and refrigerated in a plastic container at −80 °C (Haier Ultra Low Temperature Freezer Model DW-86L388, Haier, Qingdao, China) for chemical analysis. To calculate the loin eye area, the tenth rib surface area of the LTL muscle was used as described by Santos et al. [27]. The points were counted on a 1 cm^2^ plastic grid (PCGP 1 cm^2^) made of graph paper and a duplicate transparent plastic sheet. The total area was calculated by adding the squares.

### 2.5. Evaluation of Meat Quality

Slices of the LTL muscle were cut at a thickness of 2.54 cm. All samples were then vacuum sealed and stored at a temperature of −20 °C until further investigation (Haier Deep freezer Model DW-40L262, Heier, Qingdao, China). Proximate analysis methods were used to evaluate the moisture, ash, crude protein (CP), and intramuscular fat content of the LTL muscle [28]. The pork color was determined 48 h after death at three locations across the surface area of the LTL muscle after one hour of blooming time. The lightness (L*), redness (a*), and brightness (b*) values were determined using a Chroma Meter CR-400 (Minolta Camera Co., Ltd., Osaka, Japan). The instrument was calibrated with a white tile particular to the machine using a D65 illuminant, a 2° observer with an 8 mm aperture, and a 2° observer [29]. At 45 min and 24 h postmortem, the pH values of the LTL muscle and semimembranosus muscles (SM) were determined using a portable pH meter combined with a pH measuring tip with an NTC-type temperature probe (Testo Model 205—Testo, Lenzkirch, Germany). The pH meter was calibrated by comparing it to a series of calibration standards throughout the pH scale, namely 4.01/7/9.21 buffers equilibrated at ambient temperature (Mettler Toledo GmbH, Schwerzenbach, Switzerland). Each carcass was cut on the right side and the LTL muscle was transferred to the laboratory. Drip loss was assessed by storing a sample of roughly 30 g of meat in a plastic bag and keeping it for 24 h at 4 °C, following which the meat was weighed [30]. The boiling loss was estimated as the proportionate weight loss of muscle suspended in a plastic bag and immersed in an 85 °C water bath (Memmert Model WNB14, Schwabach, Germany) until the sample’s internal temperature reached 72 °C, a temperature regulated directly by the thermocouple tool. The uncooked and cooked meat weights were collected to calculate the boiling loss percentage [3,30]. The LTL samples were cut into cubes (1 × 1 × 1 cm^3^) and boiled to a final internal temperature at 75 °C in a constantly boiling water bath, followed by a rapid immersion in ice-cold water to equilibrate. The texture analyzer (Model TA.XTplusC, Stable Micro Systems, Ltd., London, UK) equipped with a Warner–Bratzler shear device was used to measured shear forces of meat. The analyses were performed by shearing perpendicular to the core’s long axis and the shear force value was recorded as the peak force of the curve [31]. To measure the marbling score values, one technician performed the measurement according to the reference standard of the National Pork Producers’ Council (NPPC) photographic scale (from 1 = devoid to 10 = abundant) [32].

### 2.6. Malondialdehyde Assay

The malondialdehyde (MDA) was analyzed following Bergamo et al. [33]. Briefly, pork aliquots (2 g) were placed in centrifuge tubes and homogenized (IKA T18D Ultra Turrax, Staufen, Germany) in 4.75 mL water and 0.25 mL ethanolic butylated hydroxytoluene (BHT) solutions, respectively. Aliquots of homogenate (500 μL) were added to 500 μL of ice-cold 10% trichloroacetic acid (TCA). After vigorously mixing the samples for 3 min, they were centrifuged for 5 min at room temperature (RT) to remove the proteins (10,000× *g*). Then, 300 μL aliquots of the sample supernatant were taken, and 700 μL of thiobarbituric acid (TBA) mix was added. After degassing, the mixtures were incubated at 90 °C for 30 min. After cooling, the samples were centrifuged using a Beckman Coulter centrifuge (Model Allegra^®^ X-22R, Indianapolis, IN, USA) for 5 min, 10,000× *g* at RT to remove particulate matter. Finally, aliquots of the sample (20 μL) were injected into the HPLC instrument. The samples were analyzed using an Agilent 1220 Infinity II liquid chromatography system coupled to an Agilent 1260 Infinity FLD spectra fluorescence detector (Agilent Technologies, Santa Clara, CA, USA). The samples were separated using a Restek Ultra Aqueous C18 reverse-phase column (250 × 4.6 mm, 5 mm, Bellefonte, PA, USA). At a flow rate of 1.0 mL/min, the mobile phase was composed of 2.5 mM sodium phosphate buffer (pH 7.0) and acetonitrile (50:50 *v*/*v*). The excitation and emission wavelengths of the fluorescence detector were adjusted to 515 nm and 543 nm, respectively. The total run time was 5 min per sample.

### 2.7. Fatty Acid Analysis

The fatty acid (FA) profile of feed, backfat, and LTL muscle was determined according to Pothakam et al. [34]. The lipids were extracted using the Soxhlet method (method 920.39) from the diet, backfat, and LTL muscle. Fatty acid methyl esters (FAMEs) were prepared following the description of Morrisson and Smith [35]. The Shimadzu GC-2030 gas chromatograph (Kyoto, Japan) was used to determine fatty acid profile. The samples were separated using a wall-coated fused wax capillary column (0.25 mm × 100 m × 0.25 μm, RT-2560, RESTEK, Bellefonte, PA, USA). As a carrier gas, helium was used. Temperatures in the injector were maintained at 250 °C. The oven temperature program was increased from 50 to 220 °C at a rate of 10 °C/min and maintained for 35 min, then increased from 200 to 230 °C at a rate of 5 °C/min and maintained for 20 min. The injection volume was 1 μL and the temperature of the flame ionization detector was set to 250 °C. Lab Solution was used to process the chromatograms (Shimadzu, Kyoto, Japan). The samples were identified by matching the retention times of their peaks to those of the FAME mixture standard (Food Industry Fame Mix, RESTEK, Bellefonte, PA, USA). Individual fatty acids were quantified in terms of g/100 g of total fatty acids [34,36].

### 2.8. Statistical Analyses

The experiment was conducted using a completely randomized design (CRD) with the pig breed, age, weight, and gender as fixed factors. The sample size for this experiment was determined using the 3Rs principle and the F-test one-way analysis of variance (ANOVA) model in G*Power-2 software, version 3.1.9.3 (Heinrich Heine Universität Düsseldorf, Düsseldorf, Germany). The general linear model (GLM) was used to analyze the pig performance, including initial weight, final weight, ADFI, ADG, and FCR of pigs with dietary treatments as the fixed effect; the significant level considered was *p* < 0.05. In addition, carcass characteristics, chemical composition, and meat qualities of pigs given different dietary treatments were investigated in same model for significant differences between groups (*p* < 0.05). The MDA content was calculated using the standard linear regression equation. GLM was used to analyze the mean of these at a significance level of *p* < 0.05. Similarly, fatty acid compositions of feed, backfat, and loin meat were calculated using standard curve regression (accepted r^2^ of standard curve > 0.99), and the average of each fatty acid value was tested statistically using GLM, and values were considered significant at *p* < 0.05. All the statistical analyses in this study were conducted using IBM SPSS Statistics 23 for Windows software (IBM Corp., Armonk, NY, USA), and Tukey’s multiple comparison test was performed to estimate mean differences between treatment groups.

## 3. Results

### 3.1. Productive Performance

The productive performance of finishing crossbred pigs fed a PC supplement in a low-lysine diet is presented in Table 3. The final weight, ADG, and FCR were found to be within a range of 105.33–107.21 kg, 0.70–0.80 kg/d, and 3.98–3.91, respectively. However, the inclusion of a PC supplement in the low-lysine diet did not result in significant difference between the treatments for the initial weight, final weight, ADFI, ADG, and FCR (*p* > 0.05).

### 3.2. Carcass Characteristics

PC supplementation in a low-lysine diet had no effect on swine carcass characteristics such as slaughter weight, dressing percentage, hot carcass weight, chilled carcass weight, carcass length, and backfat thickness (*p* > 0.05), which is illustrated in Table 4. By comparison, T3 had a significantly higher proportion of ham at primal cutting than T1 (30.38 vs. 28.69 percent). As a result, the loin proportion of PC supplementation groups was significantly greater than that of the control group at primal cutting (*p* < 0.05).

### 3.3. Meat Quality and Lipid Peroxidation

**Figure 1:**
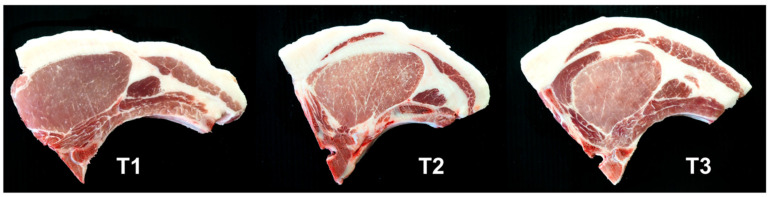
The illustration showed the marbling characteristics of pork loin from finishing crossbred pigs fed a low-lysine diet supplemented with perilla cake. T1, 0% perilla cake (PC); T2, 2.5% PC supplementation; T3, 4.5% PC supplementation.

**Figure 2:**
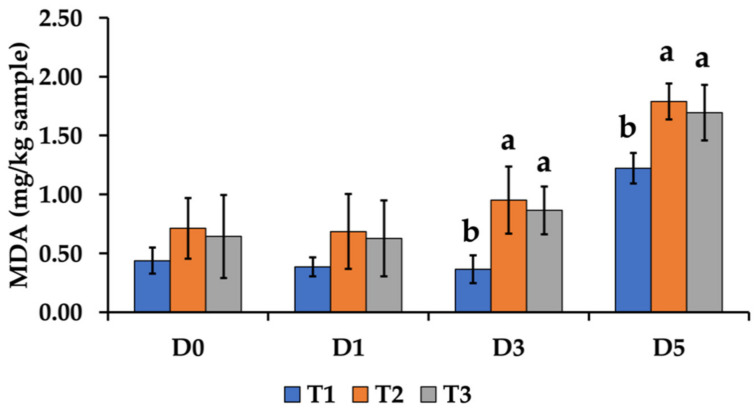
The malondialdehyde (MDA) content in *longissimus thoracis et lumborum* muscle from finishing crossbred pigs fed a low-lysine diet supplemented with perilla cake. ^a, b^ Different letters within the same row indicate a statistically significant difference (*p* < 0.05). T1, 0% perilla cake (PC); T2, 2.5% PC supplementation; T3, 4.5% PC supplementation; SEM, standard error of the mean.

The meat quality of the crossbred pig after PC supplementation in a low-lysine diet is shown in Table 5. Dietary inclusion did not affect the chemical compositions such as moisture and CP. Whereas LTL with T2 and T3 exhibited significantly higher IMF levels than T1 (4.15, 4.09 vs. 3.14%, respectively). The marbling score demonstrated the same result as the IMF, indicating that dietary PC supplementation in a low-lysine diet resulted in a significantly higher marbling score than the control group (*p* < 0.05). Figure 1 illustrates the marbling properties of LTL muscle. Additionally, we discovered that PC supplementation and reduced lysine level in the diet resulted in lower boiling loss and shear force values compared to the control group (*p* < 0.05). However, there was no significant difference in loin eye area, drip loss, color, and pH at 45 min and 24 h (*p* > 0.05). Lipid peroxidation was analyzed in this study during MDA production. On day 0 and day 1, the inclusion of PC supplementation in the low-lysine diet groups had no significant effect on the outcome compared to the control group (Figure 2). MDA levels were significantly higher with T2 and T3 on days 3 and 5 compared to T1 (*p* < 0.05). After day 3 and 5, MDA levels were significantly higher with T2 and T3 compared to T1 (*p* < 0.05).

### 3.4. Fatty Acid Profile in Backfat and LTL Muscle

We evaluated the fatty acid profiles of finishing crossbred pigs’ backfat and LTL muscle to investigate the effect of the PC supplement in a low-lysine diet. We discovered that supplementing pigs’ diets with PC had no effect on total saturated fatty acids (SFAs), total monounsaturated fatty acids (MUFAs), or the MUFA/SFA ratio in backfat (Table 6). However, the pigs fed a PC supplement in low-lysine diet had significantly higher levels of fatty acids in total polyunsaturated fatty acid (PUFA) categories except C20:3n-6, resulting in significantly higher PUFA levels than those of the control group (*p* < 0.05). In LTL muscle, the SFA content was not significantly different among groups (Table 7). It was interesting that LTL muscle had significantly higher MUFAs and PUFAs in T3 than those of the control group (*p* < 0.05). Indeed, we observed that the C18:3n-3 content in LTL muscle was significantly increased when PC was supplemented in the low-lysine diet groups. Moreover, PC supplementation in a low-lysine content diet significantly increased n-3 PUFA in both tissues, which led to a significant decrease in the n-6/n-3 ratio (*p* < 0.05).

## 4. Discussion

Pork production is critical in the modern world as a protein source. Historically, there was a strong emphasis on fat reduction in the breeding strategy for pigs as consumers demanded leaner pork, and the carcass value was largely determined by its backfat depth [11]. Consumer preferences have shifted in recent years, with an emphasis on high-quality pork. Intramuscular fat is one of the indicators for determining pork quality. The increase in the IMF content of *longissimus* muscle improved the meat tenderness and palatability for consumers purchasing pork loin [7]. The critical factor in increasing the IMF content of the pigs’ *longissimus* muscles is nutritional modification, particularly the impact of dietary lysine levels. Consequently, we designed the experiment to reduce the quantity of lysine in the diet of late-phase pigs by roughly 30% relative to the recommended dietary requirement for pigs [23], with the goal to improve IMF in the *longissimus* muscles of finishing crossbred pigs. Additionally, we supplemented PC at 2.5 and 4.5 percent in the pigs’ diet with the assumption that it would increase the fat quality in pigs. We found that the productive performance, including ADFI, ADG, and FCR, did not different significantly from the control group (*p* > 0.05). The was in agreement with previous studies showing that the performance of finishing pigs was not affected by lysine deficiency in the diet [7,11]. Our result has answered the concern that the decrease in dietary lysine may affect the productive performance of pigs. In terms of carcass features, the dietary lysine shortage and PC supplementation in the diet had no effect on carcass percentage, hot or chilled carcass weight, carcass length, or backfat thickness. However, there is exemption on ham and loin part in primal cutting. This result indicated that the ham and loin in the PC-supplemented low-lysine diet groups were significantly higher than those of the control group. This result contrasts with those of Palma-Granados et al. [37], who observed that lysine deficiency resulted in reduced carcass components, most notably ham and loin.

The impact of PC supplementation in a low-lysine diet resulted in significantly increased IMF and marbling score in crossbred pigs’ *longissimus* muscles. Several studies have confirmed that a lower lysine content in the diet is associated with a higher IMF content in pork [7,11,37]. In porcine muscle, a low-lysine diet increases the gene expression of glucose transporter protein 4 and peroxisome proliferator-activated receptor gamma (PPAR-γ), which is associated with increased mitochondrial oxidative enzyme activity [11]. The PPAR-γ gene plays a central role in the regulation of adipogenesis, a process by which fibroblast-like preadipocytes differentiate into mature adipocytes [38,39]. Moreover, the activity of mitochondrial oxidative enzymes is positively correlated with the amount of IMF in bovine muscles [11,40]. Additionally, we hypothesized that the rise in IMF in lean pork was also related to PC supplementation. Several studies indicated that dietary fat sources for pigs could increase the fatty acid content and IMF of the pork [41,42,43]. Cold-pressed perilla seed oil yields a by-product known as PC, which is high in protein (31.54%) and crude fat (10.52%). Typically, it is utilized as a source of protein in animal feeds. However, due to its high EE content and high alpha-linolenic acid content, it was added as a functional feedstuff for enhancing the fatty acid composition of animal products [18,20]. A recent study demonstrated that supplementation with perilla seed extract significantly increased the IMF content of fattening cattle’s *longissimus* muscle. Additionally, it greatly improved the activity of fatty acid accumulation enzymes, such as fatty acid synthetase and acetyl-coenzyme A carboxylase, and upregulated the PPAR-γ gene expression in fattening cattle’s *longissimus* muscle [44]. Moreover, our results demonstrated that the substantial fat deposition in the LTL muscle of pigs receiving PC supplementation in the low-lysine diet groups resulted in a much greater marbling score than those of the control group. This is consistent with earlier research demonstrating the positive correlation of IMF content on the marbling score in *longissimus* muscle [7,45,46]. The supplementation of PC in finishing pigs fed low-lysine diet significantly decreased boiling loss in pork when compared to the control group. Drip loss and boiling loss are presented in this research as measures of the water-holding capacity (WHC) of meat. The WHC influences the juiciness of cooked meats, which may affect consumers’ perceptions of tenderness [47,48]. In this investigation, there was no difference in drip loss across the groups, which was in agreement with Witte et al. [49], who showed that the dietary differential levels of lysine did not affect drip loss. One of the cooking loss parameters examined in this research was boiling loss. It was discovered that the control group’s boiling loss was significantly greater than that in the PC supplemented low-lysine diet groups. Aaslyng et al. [50] reported that meat with a low WHC (high drip loss, high thawing loss, and high internal reflection) and a low pH had a larger cooking loss, but meat with a medium or high WHC or pH had no impact. Additionally, they discovered that meat with a low IMF content had a significantly higher cooking loss at 80 °C center temperature than the meat with a high IMF level [50]. In this study, it was found that the pigs receiving PC supplementation in the low-lysine diet had a significantly lower shear force value than that in the control group. The shear force technique was utilized to determine the tenderness of meat when the IMF was increased in pig loin muscle. Our findings agreed with Ramsey et al. [51] and Jankowiak et al. [46] who reported that the meat with a high IMF content had a lower shear force value and was more tender than meat with a low IMF content.

Meat lipid peroxidation is one of the characteristics to consider when developing high-fat meat. Malondialdehyde (MDA) is an ubiquitous aldehyde generated during secondary lipid oxidation and is a widely used oxidation indicator, resulting in an increase in plasma and tissue levels of thiobarbituric acid reactive substances (TBARS) [52]. Our result show that the supplementation of PC in a low-lysine diet significantly affected MDA content in LDL muscle from day 3. Therefore, we hypothesized that it was related to the high IMF content and fatty acid composition of the meat. However, Chaiwang et al. [3] explained that the composition of fat was more important than the quantity of fat in meat, as the susceptibility of muscular lipids to peroxidation is dependent on the amount of polyunsaturated fatty acids. This was consistent with our results showing that supplementation of PC to a low-protein diet had a significant effect on the high PUFA composition of LTL muscle. According to Reitznerová et al. [52], an increase in the MDA value was correlated with an increase in the lipid content; thus, increasing FFA levels in samples resulted in an increase in the MDA content. Furthermore, we added PC to a low-lysine diet in this study to improve the quality of fatty acids in pork. The fatty acid composition of animal products is regulated by both fatty acid production in animal tissues and the lipids included in the feedstuffs ingested by animals [53]. By integrating a suitable fat source into the diet, it was feasible to modify the fatty acid composition and relationship between n-6 and n-3 fatty acids in pig tissues [20,54]. In this study, supplementation of PC to a low-lysine diet contributed to a significant increase in ALA and PUFA levels in the investigated tissues, whereas the ratio of n-6 to n-3 was considerably lower in the groups of PC supplementation. The result agreed with the report of Arjin et al. [20], who explained that the increase in PUFA and ALA levels while the n-6/n-3 ratio decreased in abdominal fat, backfat, and *longissimus* muscle of growing pigs, was affected by PC supplementation in the diet. PC is used as an oily plant that contains high levels of PUFAs, especially ALA (55.97%) [18]. As a result, we hypothesize that this is a possible explanation for the high ALA content of tissues. However, ALA deposition is dependent on a variety of parameters, including diet and tissue type. Moreover, the PUFA/SFA ratio was significantly higher in the PC-supplemented low-lysine diet groups in backfat and LTL muscle. Genetics had the greatest influence on the PUFA/SFA ratio, followed by nutrition (mostly the animal’s overall fat and intramuscular fat content) [55]. Nonetheless, only T3 in LTL muscle had a higher PUFA/SFA ratio than that recommended by the UK Department of Health [56], which is a minimum PUFA/SFA ratio of 0.4 in pork. The increased n-3 fatty acid content in the pigs affected by the supplementation of PC in the low-lysine diet leads to a lower n-6/n-3 ratio. In pig tissues, the fatty acid composition, PUFA/SFA, and n-6/n-3 ratios are greatly impacted by the fat sources in the diet [20,57]. In this study, PC supplementation resulted in a high amount of ALA in the food, which may have an effect on the fatty acid composition of pigs, especially the ALA level. ALA is a member of the n-3 (omega-3) fatty acids that play an important role in mitigating the risk of cardiovascular diseases. Therefore, improving the pork quality by reducing lysine level by supplementation with high fat content feedstuffs such as PC is an interesting strategy to create functional meat that is highly palatable and has a high-quality fatty acid profile that meets modern consumer expectations.

## 5. Conclusions

It may be concluded that supplementing perilla cake in a low-lysine diet improved meat quality, particularly intramuscular fat, and marbling score in finishing crossbred pigs’ LTL muscle. Simultaneously, supplementing this ingredient into the finishing pigs’ feed altered the composition of fatty acids in LTL muscle and backfat by improving the polyunsaturated fatty acid content, particularly ALA, as well as the PUFA/SFA, n-3, and n-6/n-3 ratios in these tissues. Perilla cake has the potential to be used in pig diets to improve the quality of pork and meet customer expectations for n-3 fatty acids in pigs. The mechanism by which perilla cake combined with a low-lysine content in the diet alters fatty acid deposition in pigs requires further investigation.

## Figures and Tables

**Table 1:** Experimental diet formulations and chemical components (dry matter basis).

Items	PC	T1	T2	T3
*Ingredient* (%)				
Maize		41.00	41.00	41.00
Broken rice		41.00	41.00	41.00
Rice bran		4.50	5.00	5.00
Perilla meal		0.00	2.50	4.50
Soybean meal (44%)		8.50	6.00	4.00
Fish meal (58%)		2.00	2.00	2.00
Lard		0.50	0.00	0.00
Dicalcium phosphate		2.00	2.00	2.00
Salt		0.25	0.25	0.25
Premix a		0.25	0.25	0.25
Total		100	100	100
*Chemical composition*				
Dry matter, %	90.12	88.09	88.22	88.29
Crude protein, %	31.60	12.20	12.05	12.08
Ether extract, %	10.56	5.23	5.53	5.87
Ash, %	6.14	6.21	6.66	6.97
Crude fiber, %	24.14	3.01	4.03	4.50
Gross energy, cal/g	4658	3358	3400	3367
Lysine, %	19.50 *	0.60	0.42	0.42
Methionine,%	10.88 *	0.46	0.36	0.37

Mean of triplicate (*n* = 3) (chemical composition). PC, ground perilla cake; T1, 0% PC; T2, 2.5% PC; T3, 4.5% PC supplementation. ^a^ Each kg of vitamin premix contains 12,000 U vitamin A, 4500 U vitamin D3, 70 U vitamin E, 3.5 mg vitamin K, 3 mg vitamin B1, 7.5 mg vitamin B2, 30 mg vitamin B3, 65 mg vitamin B5, 4.3 mg vitamin B6, 2 mg vitamin B9, 0.025 mg vitamin B12, 0.3 mg biotin, and 800 mg choline chloride. * PC lysine and methionine units are mg/g.

**Table 2:** Compositions of fatty acids in three dietary treatments fed to the finishing crossbred pigs (g/100 g of total fatty acid).

Fatty Acids	T1	T2	T3	SEM	*p*-Value
*Saturated fatty acid (SFA)*					
C14:0	0.33 ^c^	0.54 ^b^	0.61 ^a^	0.052	0.000
C16:0	15.97 ^c^	18.87 ^a^	18.20 ^a^	0.573	0.017
C17:0	0.19 ^b^	0.26 ^a^	0.27 ^a^	0.014	0.001
C18:0	3.43 ^c^	4.82 ^b^	4.94 ^a^	0.306	0.000
C20:0	0.91 ^a^	0.92 ^a^	0.86 ^b^	0.013	0.012
C22:0	0.68 ^a^	0.51 ^b^	0.43 ^c^	0.042	0.000
C23:0	0.24 ^b^	0.23 ^b^	0.28 ^a^	0.009	0.003
*Monounsaturated fatty acid (MUFA)*					
C16:1n-7	0.54 ^c^	0.73 ^b^	0.80 ^a^	0.048	0.001
C17:1n-8	0.05	0.05	0.06	0.003	0.465
C18:1n-9	41.27 ^a^	36.25 ^b^	35.95 ^b^	1.099	0.001
C20:1n-9	0.43	0.38	0.39	0.135	0.299
*Polyunsaturated fatty acid (PUFA)*					
C18:2n-6	32.56 ^a^	31.16 ^ab^	29.57 ^b^	0.566	0.018
C18:3n-6	0.03 ^b^	0.05 ^a^	0.07 ^a^	0.008	0.010
C18:3n-3	1.26 ^c^	3.17 ^b^	5.73 ^a^	0.834	0.007
C20:2n-6	0.58	0.76	0.30	0.095	0.098
C20:3n-6	0.03	0.02	0.02	0.004	0.306
C20:5n-3	0.33 ^a^	0.28 ^b^	0.34 ^a^	0.013	0.003
C22:6n-3	1.15 ^b^	0.98 ^c^	1.18 ^a^	0.039	0.000
ΣSFA	21.76 ^c^	26.14 ^a^	25.57 ^a^	0.884	0.004
ΣMUFA	42.29 ^a^	37.40 ^b^	37.19 ^b^	1.060	0.002
ΣPUFA	35.95 ^b^	36.43 ^ab^	37.22 ^a^	0.243	0.016
ΣMUFA/ΣSFA	1.94 ^a^	1.43 ^b^	1.45 ^b^	0.106	0.002
ΣPUFA/ΣSFA	1.65 ^a^	1.39 ^b^	1.45 ^b^	0.050	0.008
n-6	32.62 ^a^	31.24 ^ab^	29.67 ^b^	0.558	0.018
n-3	2.74 ^b^	4.44 ^b^	7.25 ^a^	0.847	0.007
n-6/n-3	11.89 ^a^	7.03 ^b^	4.12 ^c^	1.436	0.000

^a, b, c^ Different superscripts within the same row show a statistically significant difference (*p* < 0.05). T1, 0% perilla cake (PC); T2, 2.5% PC supplementation; T3, 4.5% PC supplementation; SEM, standard error of the mean.

**Table 3:** Productive performance of a finishing crossbred pigs fed a low-lysine diet supplemented with perilla cake.

Items	T1	T2	T3	SEM	*p*-Value
Initial weight, kg	73.57	73.65	74.52	0.741	0.867
Final weight, kg	107.21	105.92	105.33	0.664	0.526
ADFI, kg/day	3.19	2.98	2.90	0.068	0.195
ADG, kg/day	0.80	0.77	0.73	0.014	0.135
FCR	3.98	3.91	3.98	0.104	0.950

T1, 0% perilla cake (PC); T2, 2.5% PC supplementation; T3, 4.5% PC supplementation; SEM, standard error of the mean.

**Table 4:** Carcasses characteristic of a finishing crossbred pigs fed a low-lysine diet supplemented with perilla cake.

Items	T1	T2	T3	SEM	*p*-Value
Slaughter weight (kg)	107.21	105.92	105.33	0.664	0.526
Dressing percentage (%)	74.47	75.89	76.63	0.761	0.544
Hot carcass weight, kg	79.83	80.36	80.75	0.822	0.915
Chilled carcass weight, kg	77.86	78.78	78.92	0.734	0.828
Carcass length, cm	93.57	93.00	92.00	0.684	0.673
Backfat thickness, cm	3.51	3.72	3.54	0.129	0.787
Primal cutting (%)					
Shoulder	34.42	33.38	32.38	0.536	0.337
Ham	28.69 ^b^	29.84 ^ab^	30.38 ^a^	0.263	0.022
Belly	16.11	15.03	14.09	0.446	0.203
Loin	16.15 ^b^	17.96 ^a^	17.35 ^a^	0.310	0.019

^a, b^ Different superscripts within the same row indicate a statistically significant difference (*p* < 0.05). T1, 0% perilla cake (PC); T2, 2.5% PC supplementation; T3, 4.5% PC supplementation; SEM, standard error of the mean.

**Table 5:** Meat quality of a finishing crossbred pigs fed a low-lysine diet supplemented with perilla cake.

Items	T1	T2	T3	SEM	*p*-Value
Chemical compositions (%)					
Moisture	73.01	72.21	72.60	0.199	0.249
CP	18.96	18.89	20.10	0.262	0.117
Ash	1.34 ^a^	1.27 ^b^	1.23 ^b^	0.016	0.012
Intramuscular fat (g/100 g) ^‡^	3.14 ^b^	4.15 ^a^	4.09 ^a^	0.096	0.000
Marbling score	3.39 ^b^	4.26 ^a^	4.17 ^a^	0.094	0.000
Loin eye area (cm^2^)	48.92	49.61	49.10	0.486	0.909
Drip loss (%)	5.07	4.69	4.10	0.408	0.654
Boiling loss (%)	27.61 ^a^	21.78 ^b^	22.43 ^b^	0.903	0.006
Shear force (N/cm^2^)	54.50 ^a^	39.13 ^b^	41.89 ^b^	2.507	0.015
Color (24 h)					
L*	48.81	48.92	49.35	0.618	0.943
a*	8.09	7.52	8.14	0.486	0.741
b*	4.49	4.59	4.09	0.573	0.942
pH (45 min)					
LTL	6.26	6.25	6.23	0.034	0.939
SM	6.26	6.47	6.22	0.048	0.067
pH (24 h)					
LTL	5.77	5.76	5.83	0.048	0.864
SM	5.94	5.90	5.93	0.094	0.989

^a, b^ Different superscripts within the same row show a statistically significant difference (*p* < 0.05). ^‡^ IMF is expressed as g of lipid in 100 g of *longissimus thoracis et lumborum* (LTL) muscle tissue. T1, 0% perilla cake (PC); T2, 2.5% PC supplementation; T3, 4.5% PC supplementation; SM, *semimembranosus* muscle; SEM, standard error of the mean. Data for the chemical composition represent the mean of triplicates.

**Table 6:** Fatty acid composition of backfat from finishing crossbred pigs fed a low-lysine diet supplemented with perilla cake (g/100 g of total fatty acids).

Fatty Acids	T1	T2	T3	SEM	*p*-Value
*Saturated fatty acid (SFA)*					
C14:0	1.29	1.32	1.25	0.040	0.789
C16:0	30.07	25.70	25.26	1.310	0.253
C17:0	0.15	0.23	0.14	0.030	0.415
C18:0	12.45	13.15	12.92	0.263	0.537
C20:0	0.24	0.25	0.45	0.061	0.295
C22:0	0.03 ^b^	0.04 ^ab^	0.05 ^a^	0.002	0.036
C23:0	0.13	0.13	0.14	0.005	0.823
*Monounsaturated fatty acid (MUFA)*					
C16:1n-7	2.08	1.93	1.73	0.075	0.177
C17:1n-8	0.15	0.14	0.14	0004	0.255
C18:1n-9	42.55	45.04	44.71	0.793	0.376
C20:1n-9	0.77 ^b^	1.03 ^a^	0.80 ^b^	0.036	0.003
*Polyunsaturated acid (PUFA)*					
C18:2n-6	9.12 ^b^	9.53 ^ab^	10.76 ^a^	0.245	0.017
C18:3n-6	0.02 ^b^	0.02 ^ab^	0.03 ^a^	0.001	0.040
C18:3n-3	0.55 ^c^	1.47 ^b^	2.08 ^a^	0.109	0.000
C20:2n-6	0.42 ^b^	0.55 ^a^	0.54 ^a^	0.016	0.000
C20:3n-6	0.05	0.06	0.06	0.001	0.110
C20:5n-3	0.01 ^b^	0.01 ^b^	0.02 ^a^	0.001	0.004
C22:6n-3	0.07 ^b^	0.08 ^ab^	0.09 ^a^	0.002	0.004
ΣSFA	44.38	40.81	40.21	1.053	0.218
ΣMUFA	45.56	48.13	47.38	0.846	0.437
ΣPUFA	10.21 ^c^	11.73 ^b^	13.57 ^a^	0.325	0.000
ΣMUFA/ΣSFA	1.08	1.17	1.18	0.026	0.229
ΣPUFA/ΣSFA	0.24 ^b^	0.29 ^b^	0.34 ^a^	0.008	0.000
n-6	10.13 ^b^	10.17 ^b^	11.30 ^a^	0.173	0.007
n-3	0.64 ^c^	1.56 ^b^	2.16 ^a^	0.110	0.000
n-6/n-3	15.83 ^a^	6.51 ^b^	5.23 ^b^	1.025	0.000

^a, b, c^ Different superscript letters within the same row indicate a statistically significant difference (*p* < 0.05). T1, 0% perilla cake (PC); T2, 2.5% PC supplementation; T3, 4.5% PC supplementation; SEM, standard error of the mean.

**Table 7:** Fatty acid composition of *longissimus thoracis et lumborum* muscle from finishing crossbred pigs fed a low-lysine diet supplemented with perilla cake (g/100 g of total fatty acids).

Fatty Acids	T1	T2	T3	SEM	*p*-Value
*Saturated fatty acid (SFA)*					
C14:0	1.09 ^b^	1.22 ^a^	1.28 ^a^	0.020	0.000
C16:0	17.38 ^b^	18.25 ^a^	17.71 ^b^	0.100	0.000
C17:0	1.05 ^a^	0.70 ^b^	0.88 ^ab^	0.042	0.001
C18:0	16.31	15.35	15.75	0.283	0.369
C20:0	0.32	0.37	0.37	0.048	0.882
C22:0	0.21 ^a^	0.14 ^b^	0.08 ^c^	0.011	0.000
C23:0	2.81 ^a^	1.84 ^b^	1.98 ^b^	0.108	0.000
*Monounsaturated fatty acid (MUFA)*					
C16:1n-7	4.25 ^b^	6.15 ^a^	6.81 ^a^	0.294	0.000
C17:1n-8	0.52	0.40	0.47	0.022	0.078
C18:1n-9	36.76 ^b^	38.40 ^ab^	38.97 ^a^	0.317	0.009
C20:1n-9	1.34	1.16	1.43	0.047	0.069
*Polyunsaturated acid (PUFA)*					
C18:2n-6	12.75	11.69	13.17	0.302	0.142
C18:3n-6	0.12 ^a^	0.16 ^a^	0.07 ^b^	0.010	0.000
C18:3n-3	1.53 ^b^	3.52 ^a^	3.83 ^a^	0.195	0.000
C20:2n-6	0.25 ^c^	0.40 ^b^	0.78 ^a^	0.043	0.000
C20:3n-6	0.41 ^a^	0.31 ^b^	0.29 ^b^	0.012	0.000
C20:5n-3	0.12	0.14	0.14	0.008	0.461
C22:6n-3	0.34 ^ab^	0.28 ^b^	0.42 ^a^	0.023	0.054
ΣSFA	43.87	43.44	43.06	0.197	0.209
ΣMUFA	42.87 ^b^	44.32 ^b^	46.52 ^a^	0.418	0.000
ΣPUFA	14.64 ^b^	16.49 ^ab^	18.71 ^a^	0.602	0.011
ΣMUFA/ΣSFA	0.98 ^b^	1.02 ^b^	1.08 ^a^	0.011	0.000
ΣPUFA/ΣSFA	0.33 ^b^	0.38 ^ab^	0.43 ^a^	0.014	0.006
n-6	14.86 ^a^	12.49 ^b^	14.25 ^ab^	0.334	0.012
n-3	2.01 ^b^	3.99 ^a^	4.47 ^a^	0.238	0.000
n-6/n-3	7.38 ^a^	3.13 ^b^	3.19 ^b^	0.445	0.000

^a, b, c^ Different superscripts within the same row indicate a statistically significant difference (*p* < 0.05). T1, 0% perilla cake (PC); T2, 2.5% PC supplementation; T3, 4.5% PC supplementation; SEM, standard error of the mean.

## Data Availability

Not applicable.
